# Disarming Gram-Negative Bacteria in the Fight Against Antimicrobial Resistance

**DOI:** 10.1021/acscentsci.3c01474

**Published:** 2023-12-13

**Authors:** Glen Brodie, Stuart J. Conway

**Affiliations:** Department of Chemistry & Biochemistry, University of California Los Angeles, 607 Charles E. Young Drive East, P.O. Box 951569, Los Angeles, California 90095-1569, United States

In this issue of *ACS Central Science*, Hirsch and co-workers report an innovative strategy to treat infections caused by the Gram-negative bacterium *Pseudomonas aeruginosa* by targeting its main virulence factor, LasB.^1^*P. aeruginosa* is a multidrug resistant opportunistic bacterium that is listed among the World Health Organization’s critical group of pathogens for which new antibiotics are urgently required.^2^ Antimicrobial resistance (AMR) is a significant and increasing threat to global public health that was associated with 4.95 million deaths in 2019.^3,4^ This figure could rise to over 10 million deaths per year by 2050 unless immediate action is taken.^5^ AMR has been exacerbated by the overuse and misuse of antibiotics, and new strategies for treating infections are being sought that will minimize the development of resistance.

*P. aeruginosa* causes either acute or chronic infection in people who are immunocompromised, including those with chronic obstructive pulmonary disease (COPD), cystic fibrosis, and cancer.^6^ As *P. aeruginosa* is a Gram-negative pathogen, it has an outer membrane, in addition to an inner cell wall. This makes targeting proteins in Gram-negative pathogens especially challenging, as drugs that function by interacting with targets inside the bacterium must first pass through the outer membrane *and* cell wall before they can become effective. To achieve this, medicinal chemists must develop drug molecules that possess an appropriate balance of lipophilicity, hydrophobicity, molecular weight, and stability. This challenge is one of the reasons that there is a lack of drugs to treat multidrug resistant *P. aeruginosa-*derived infections. To sidestep this issue, Hirsch and co-workers have elected to target LasB, which is an *extracellular* enzyme, bypassing the need for the drugs to enter the bacterium. By removing the need for the compounds to enter the *P. aeruginosa*, the authors were free to explore a wider range of chemical space in their study. The target of this work, LasB, is an example of a virulence factor, which are enzymes associated with microbe “infectivity” and inflicting damage on the host. Inhibiting virulence factors hinders the ability of the bacterium to infect the host, effectively “disarming” it, rendering it unable to establish an infection or to cause damage to the host. However, this inhibition does not kill the pathogen, and consequently less selection pressure is placed upon the pathogen to develop resistance against drugs targeting these proteins.

**Figure 1:**
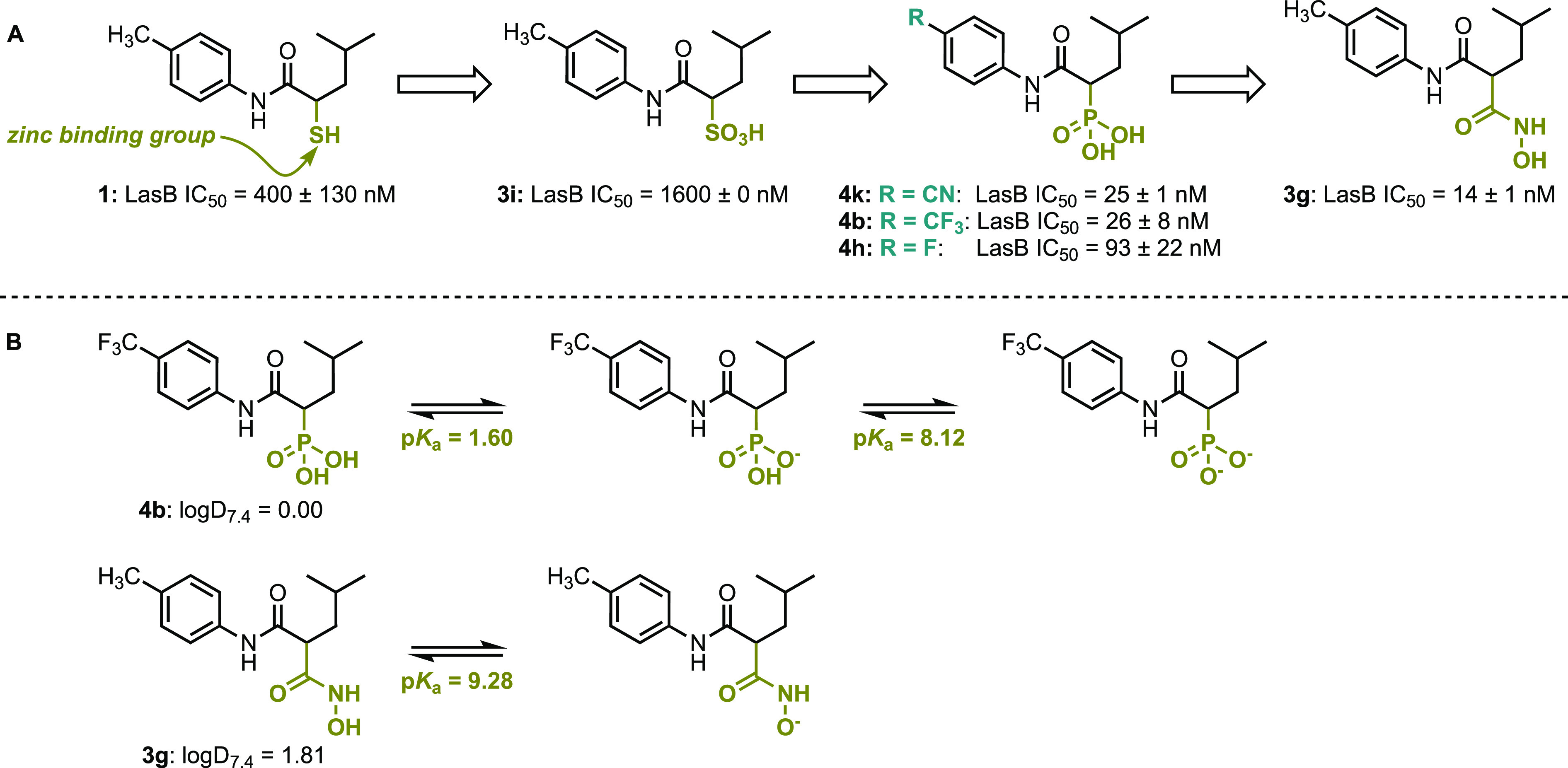
(A) The evaluation of the zinc-binding group (ZBG) to identify potent LasB inhibitors. (B) The p*K*_a_ and logD_7.4_ values for compounds **3g** and **4b**. The p*K*_a_ values were calculated using Chemicalize. The logD_7.4_ values are taken from Konstantinović et al.^1^

LasB is a Zn^2+^-dependent metalloprotease, and consequently its inhibitors contain a zinc-binding group (ZBG). The nature of this group can present challenges in terms of selectivity over other Zn^2+^-dependent enzymes and the physicochemical properties of the ZBG itself.^7^ Hirsch and co-workers had previously developed the thiol-based LasB inhibitor, **1** (Figure 1A).^8^ While this compound shows reasonably high potency against LasB, the low chemical stability of the thiol group means that this compound is not suitable for development as a therapeutic agent. Analysis of alternative ZBGs identified the hydroxamic acid **3g** and the phosphonate derivatives **4b**, **4h**, and **4k**, as highly potent LasB inhibitors.

**Figure 2:**
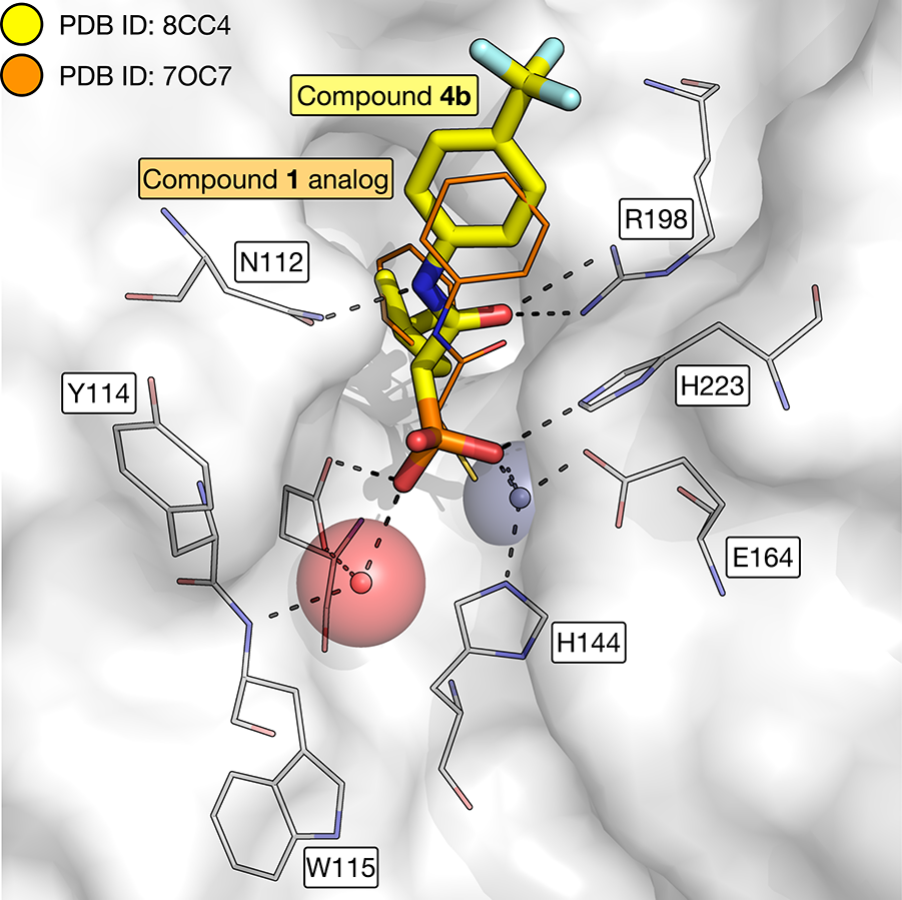
X-ray crystal structure of compound **4b** (carbon = yellow; PDB ID: 8CC4) bound to LasB (carbon = white; PDB ID: 8CC4) overlaid with the X-ray crystal structure of an analog of compound **1**, (*R*)-2-mercapto-*N*,3-diphenylpropanamide (carbon = orange; PDB ID: 7OC7) bound to LasB.

To determine the optimum ZBG for further studies, ADMET (Absorption, Distribution, Metabolism, Excretion, and Toxicity) profiling was used to compare the phosphonates and hydroxamic acid. A key criterion was permeability across a Calu-3 monolayer, and unusually, the aim was to identify compounds that have low permeability in the Calu-3 assay, as this corresponds with high retention in the lung. This low permeability minimizes systemic circulation of the drug and is an important feature of anti-pseudomonal drugs targeting the lung. The authors noted that there was a strong correlation between measured permeabilities and chromatographic lipophilicities, with the hydroxamic acid **3g** showing higher permeability than the phosphonate **4b**. The hydroxamic acid has a higher logD_7.4_ value than the phosphonate, which likely results from the differing p*K*_a_ values of these motifs. Hydroxamic acids typically have a p*K*_a_ value of around 9.3, the calculated value for **3g** is 9.28, and so they are not substantially deprotonated at physiological pH.^9^ Conversely, the p*K*_a_ value of the first phosphonate deprotonation is typically 2.4, the calculated value for **4b** is 1.60, and the p*K*_a_ value of the second deprotonation is approximately 7.5, the calculated value for **4b** is 8.12, and so phosphonates are at least monoanionic at pH 7.4 (Figure 1B).^10^ In addition, the hydroxamic acid **3g** caused some growth inhibition of the human A549 lung adenocarcinoma cell line through off-target activity. Based on these data, the phosphonates were progressed for further studies. The authors obtained an X-ray crystal structure of compound **4b** bound to LasB (Figure 2; PDB ID 8CC4), demonstrating that the phosphonate acts as the ZBG, as expected. Overlaying the structure with that of an analog of compound **1** bound to LasB (Figure 2; PDB ID: 7OC7) shows that both compounds bind in a similar manner. Interestingly, the (*R*)-enantiomer of each compound is observed in the structures, suggesting that this enantiomer binds to LasB in preference to its antipode. While it is likely that the individual enantiomers of the phosphonates would not be stable in cells, addition of a fluoride or other nonproton group at the stereogenic center might enable separation and biological evaluation of the stereoisomers.

Studies on the phosphonates in 2D A549 cells, and 3D lung organoids, treated with *P. aeruginosa* showed that the cell viability was retained even at low concentrations of the compounds. These studies also demonstrated that the mechanism of action of these compounds derives predominantly from inhibition of LasB. Work in the *Galleria mellonella* larvae infection model demonstrated that phosphonate **4b** does not have any antibacterial action itself, which is important to help avoid resistance developing, but significantly enhanced the action of the antibiotic tobramycin. Following pharmacokinetic studies in mice, a combination of the antibiotic levofloxacin and **4b** was shown to significantly reduce the number of colony-forming units in mice lungs treated with *P. aeruginosa* DSM-1117.

Overall, this is a very interesting study that provides compelling evidence for targeting the LasB virulence factor as a method to aiding *P. aeruginosa* treatment. The extracellular location of this protein makes it an attractive target in this Gram-negative bacterium, where permeability is often a key challenge in antibiotic drug development. The use of phosphonate groups in drugs is often hampered by their poor permeability, and here Hirsch and co-workers elegantly use this characteristic to their advantage, minimizing the systemic exposure of the compounds and retaining it at the key site of action in the lung. The lead compounds reported here look well-suited for further development toward inhaled drugs as treatment options for infections in cystic fibrosis and non-cystic fibrosis bronchiectasis patients.
